# Effect of trabecular architectures on the mechanical response in osteoporotic and healthy human bone

**DOI:** 10.1007/s11517-024-03134-8

**Published:** 2024-06-01

**Authors:** Chiara Bregoli, Carlo Alberto Biffi, Ausonio Tuissi, Federica Buccino

**Affiliations:** 1 National Research Council, CNR-ICMTE, Lecco, Italy; 2 Mechanical Engineering Department, Politecnico Di Milano, Milano, Italy; 3 IRCCS Istituto Ortopedico Galeazzi, Milan, Italy

**Keywords:** Human trabecular bone, Morphometric parameters, Mutual relationships, Bone mechanical behaviour, Computational mesoscale models

## Abstract

Research at the mesoscale bone trabeculae arrangement yields intriguing results that, due to their clinical resolution, can be applied in clinical field, contributing significantly to the diagnosis of bone-related diseases. While the literature offers quantitative morphometric parameters for a thorough characterization of the mesoscale bone network, there is a gap in understanding relationships among them, particularly in the context of various bone pathologies. This research aims to bridge these gaps by offering a quantitative evaluation of the interplay among morphometric parameters and mechanical response at mesoscale in osteoporotic and non-osteoporotic bones. Bone mechanical response, dependent on trabecular arrangement, is defined by apparent stiffness, computationally calculated using the Gibson-Ashby model. Key findings indicate that: (i) in addition to bone density, measured using X-ray absorptiometry, trabecular connectivity density, trabecular spacing and degree of anisotropy are crucial parameters for characterize osteoporosis state; (ii) apparent stiffness values exhibit strong correlations with bone density and connectivity density; (iii) connectivity density and degree of anisotropy result the best predictors of mechanical response. Despite the inherent heterogeneity in bone structure, suggesting the potential benefit of a larger sample size in the future, this approach presents a valuable method to enhance discrimination between osteoporotic and non-osteoporotic samples.

**Graphical Abstract:**

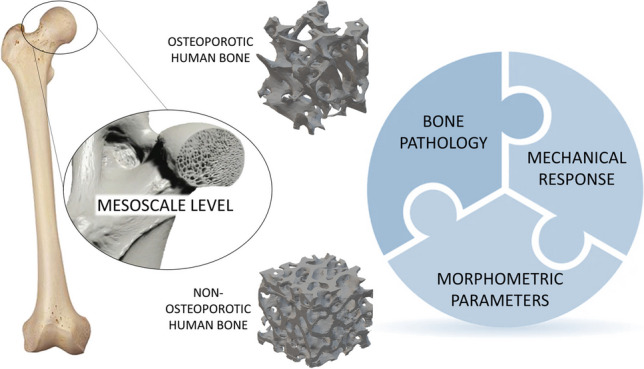

**Supplementary Information:**

The online version contains supplementary material available at 10.1007/s11517-024-03134-8.

## Introduction

Bone structure is characterized by a hierarchical organization in which macroscale, mesoscale and microscale level of structures interact each other affecting the global mechanical performances of bone tissue [1, 2]. Despite the widespread pursuit of microscale and nanoscale advancements in recent times [3], their current impact on clinical applications is limited. This is primarily because there is a lack of suitable medical tools available in the market to directly leverage these results. On the contrary, macroscale and, especially, mesoscale tissue levels are deepened in clinic thanks to the proper technologies, such as dual X-ray absorptiometry (DXA), high resolution computed tomography (CT) and magnetic resonance imaging (MRI) [4–6]. In light of the potential clinical implications, researchers are diligently directing their efforts towards augmenting the comprehension of the morphological arrangement and mechanical response of bone tissue at the mesoscale. This emphasis arises from the recognition of the significant impact that investigations on mesoscale bone tissue may exert in clinical settings. The burgeoning interest in elucidating the architectural arrangement of mesoscale human bone persists, driven by the ongoing alterations in trabecular structure observed throughout the aging process and in pathological conditions. [5, 7]. These studies on bone mesoscale structure aim to enhance both the definition of the fabric tissue properties and their relationships with global and local mechanical responses [8]. The future goal of research consists in the identification of tools, information and analysis useful for the improvement of bone disease diagnosis. Currently, DXA technique is the most widespread tool in clinic to evaluate the average bone density of human bone at mesoscale for the consequent diagnosis of bone pathologies, such as osteoporosis [9]. Concerning the clinical standards, the World Health Organization (WHO) defines the criteria to diagnose osteoporosis which include T-score < 2.5 [10]. However, it has been shown that bone density alone can hinder the correct clinical evaluation of bone [11, 12], and it may be not sufficient to diagnose osteoporosis [13].

Firstly, starting from high resolution CT and MRI of bone site [14–16], researches at mesoscale tissue level aspire to investigate additional morphometric parameters useful to optimize the evaluation of the physio-pathological state of bone [11]. Among these parameters bone density, trabecular thickness and trabecular spacing are the most studied [17, 18]. Recently, the orientation of trabeculae and their interconnectivity become useful to improve the characterization of samples with different physio-pathological conditions [12, 19–21]. In this context, imaging software industries started to develop efficient algorithms for the analysis of such relevant morphometric parameters, such as the degree of anisotropy, trabecular connectivity density, and ellipsoid index [22]. The orientation of bone trabeculae is described by the degree of anisotropy index [23–26]. Trabeculae interconnectivity is described by the connectivity density, i.e. the number of trabeculae connection per unit of volume [23]. Ellipsoid index defines the rod-like or plate-like structure, and it is considered the parameter useful to describe the transition from healthy to pathological bone conditions [27]. The aforementioned morphometric parameters, such as bone density, trabecular thickness, trabecular spacing, connectivity density, anisotropy and ellipsoid factor, are mainly investigated individually, and only few correlations among them in mainly healthy bone conditions are examined in the literature [5, 7, 12, 28]; no correlation and comparison of morphometric parameters in osteoporotic and non-osteoporotic samples is examined. A deep investigation of the mutual associations among morphometric parameters in different physio-pathological state is necessary to provide information useful into clinic.

Secondly, differences in architectural arrangement lead to difference in bone strength: anisotropy of trabecular structures, trabecular spatial orientation and trabecular thickness are hypothesised to play a crucial role in the determination of mechanical response [4]. Bone mechanical strength at mesoscale is often evaluated experimentally by means of destructive tests, such as reported by Ciarelli et al., Rieger et al. and Nikodem [11, 17, 29, 30] and finite element analysis (FEA) [28, 31, 32]. Ciarelli et al. analyse the relation between experimental maximum Young modulus and bone density [29], while Rieger et al. investigate the connection between experimental and computational Young modulus values [17]. However, both the previous studies do not consider the mutual relationships among mechanical behaviour and fabric tissue parameters, such as connectivity density and anisotropy. Nikodem [11] approaches the study of correlation between experimentally mechanical properties and few morphometric parameters, such as bone density and trabecular thickness, but, in spite of the completeness of the research, a comparison of mutual relationships among morphometric parameters is not deepened. Concerning computational analysis, despite the relevant information obtained from FEA, output of computational models are rarely explained in correlation with bone architectural arrangement [28, 31, 32].

Due to the strong interdependence between architectural arrangement at mesoscale and bone mechanical response, the analysis of architectural arrangement of bone at mesoscale will have strong impact both on clinical field and on the analysis of fracture and damage propagation in bone [33].

This work aims at shedding light on the correlations between the mesoscale features and mechanical response in osteoporotic and non-osteoporotic human bone samples. To achieve this goal, osteoporotic and non-osteoporotic samples are harvested from femoral head, observed by means of high-resolution synchrotron scans to achieve a 3D data set of each specimen. After data set post-processing, morphometric parameters are measured for each sample: bone density, trabecular thickness, trabecular spacing, anisotropy index, connectivity density and ellipsoid factor. A statistical evaluation of each morphometric parameters is carried out, and possible correlations among them are quantified. In addition to defining the parameter set, FEA analyses are performed to develop mesoscale bone models useful for describing the mechanical response of the bone by determining apparent stiffness values: Trabecular bone is considered a ‘cellular solid’ according to Gibson-Ashby model [34]. Apparent stiffness values are compared and explained in correlation with the bone morphological features at mesoscale. Firstly, the mechanical response of bone is strongly related to bone density, connectivity density and degree of anisotropy. Secondly, degree of anisotropy, connectivity density and trabecular spacing result the best morphometric parameters for the discrimination between osteoporotic and non-osteoporotic state. Finally, connectivity density and degree of anisotropy result the best predictors of mechanical response at mesoscale. Further researches are ongoing to increase the sampling size, with the aim to enforce the analysis and provide useful indicators for an improved clinical evaluation of osteoporotic and non-osteoporotic patients.

## Materials and methods

### Bone specimen preparation

**Fig. 1:**
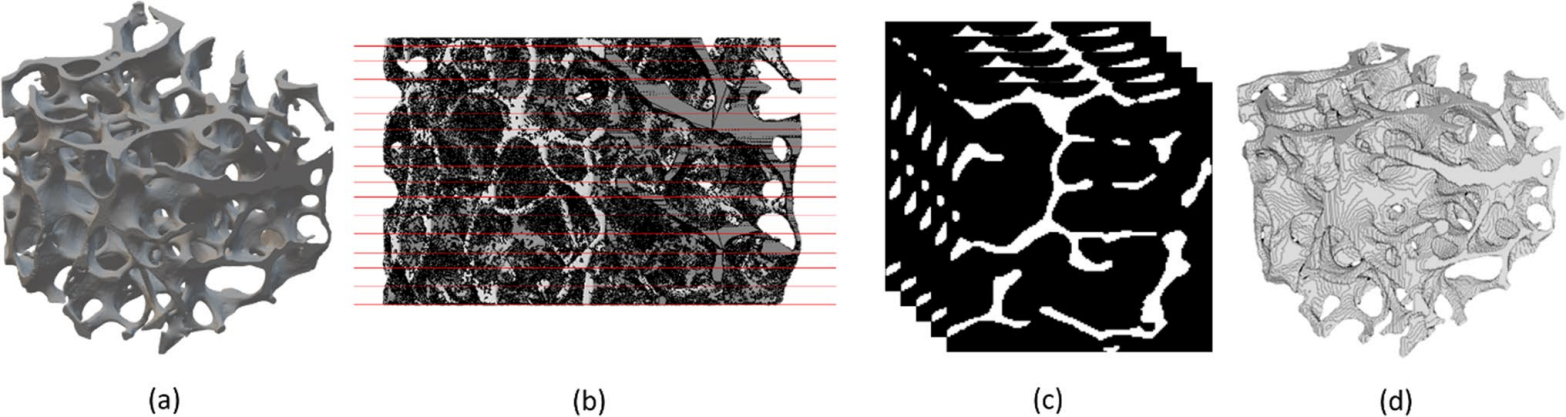
Pipeline for post-processing of stl file. A smoothed stl file of sample (**a**). Slicing procedure (**b**). Stuck of slice obtained for each sample (**c**). ImageJ 3D evaluation of the 3D data set (**d**)

Trabecular bone samples of 4 mm × 4 mm × 4 mm size [25] are harvested from femoral head of patients treated with hip arthroplasty. The femoral heads are collected prior authorization from the Ethics Committee (extension approval date: November,2023 2020, ClinicalTrials.gov ID: NCT04787679) of San Raffaele Hospital (Milano, Italy) and signed approval consent of the patients. Seven samples are collected from osteoporotic patients (OS) and seven samples from patients with no detected osteoporosis (NOS) (Fig. 1). The classification of osteoporotic and non-osteoporotic state is performed in accordance to WHO guidelines (T_score < 2.5 for osteoporotic case) [35]. Patient age, gender and clinical evaluation of each samples are summarized in Table 1. Samples are cut along the principal direction of the compressive and tensile lines [36]: The longitudinal *z*-axis of each sample coincides with the force lines direction. The cut segments are then fixed in formaldehyde to avoid bacteria contamination, stored in 70% ethanol and embedded into epoxy resin endcaps.

**Table 1:** Specific characteristic of each samples and donor which samples come from: age, gender and clinical evaluation

Femoral head sample	Patient age (years)	Gender	Clinical evaluation*
OS_1	73	Female	Osteoporosis
OS_2	90	Female	Osteoporosis
OS_3	90	Female	Osteoporosis
OS_4	76	Female	Osteoporosis
OS_5	58	Female	Osteoporosis
OS_6	83	Female	Osteoporosis
OS_7	83	Female	Osteoporosis
NOS_1	71	Female	Non-osteoporosis
NOS_2	72	Female	Non-osteoporosis
NOS_3	76	Female	Non-osteoporosis
NOS_4	74	Female	Non-osteoporosis
NOS_5	n.a	Female	Non-osteoporosis
NOS_6	46	Female	Non-osteoporosis
NOS_7	n.a	Female	Non-osteoporosis

*Clinical evaluation is based on WHO standards; *n.a.* not available

### Scanning procedure

Synchrotron scans are performed in the experimental hutch of the SYRMEP beamline (Elettra synchrotron, Trieste, Italy), as described in detail in [8, 30]. Briefly, scans are carried out using the white beam configuration in propagation-based phase-contrast modality with a sample-to-detector distance of 150 mm, and an X-ray energy of 25.6 keV obtained with a silicon foil 1.5-mm thick. Projections are recorded with a camera based on a 16-bit, water-cooled Orca Flash 4.0 sCMOS detector (2048 × 2048 pixels) coupled to a 17-µm-thick GGG scintillator screen. The synchrotron scans are performed according to the so-called half acquisition mode (i.e. an off-centre rotation over 360°, in order to almost double the width of the field of view), according to the following parameters: pixel size = 1.6 µm, exposure time = 0.1 s, number of projections = 1800 and field of view = 3.28 · 3.28 mm. The scan is performed in fly mode; for each scan, one flat field image (without the sample) and one dark field image (without X-ray illumination) are taken. For each sample, a 3D data set is collected.

### Post-processing of the scanned samples

Firstly, post-processing is performed in Matlab to obtain an stl. file of each sample [36]. Secondly, optimization of stl files is performed in 3-matic (Mimics, Materialize, Germany). Optimal post-processing parameters is set to smooth the file at microscale without losing information at the mesoscale. A smoothing factor equal to 1 is set. The noise reduction function permits to finalize optimization of the stl. file. Each object file is imported in stl software (Netfabb, Autodesk, United States) for slicing procedure. Slice thickness is set to 0.03 mm, chosen as equal to an order of magnitude less than the interested morphometric parameters, detailed in the paragraph 2.4. For each samples, a stuck of slice with a resolution of 0.03 mm × 0.03 mm × 0.03 mm is obtained. The stuck of slice is imported into BoneJ (FiJi, ImageJ, United States) [37] to proceed with the analysis of morphometric bone parameters (Fig. 1).

### Morphometric bone parameters

**Fig. 2:**
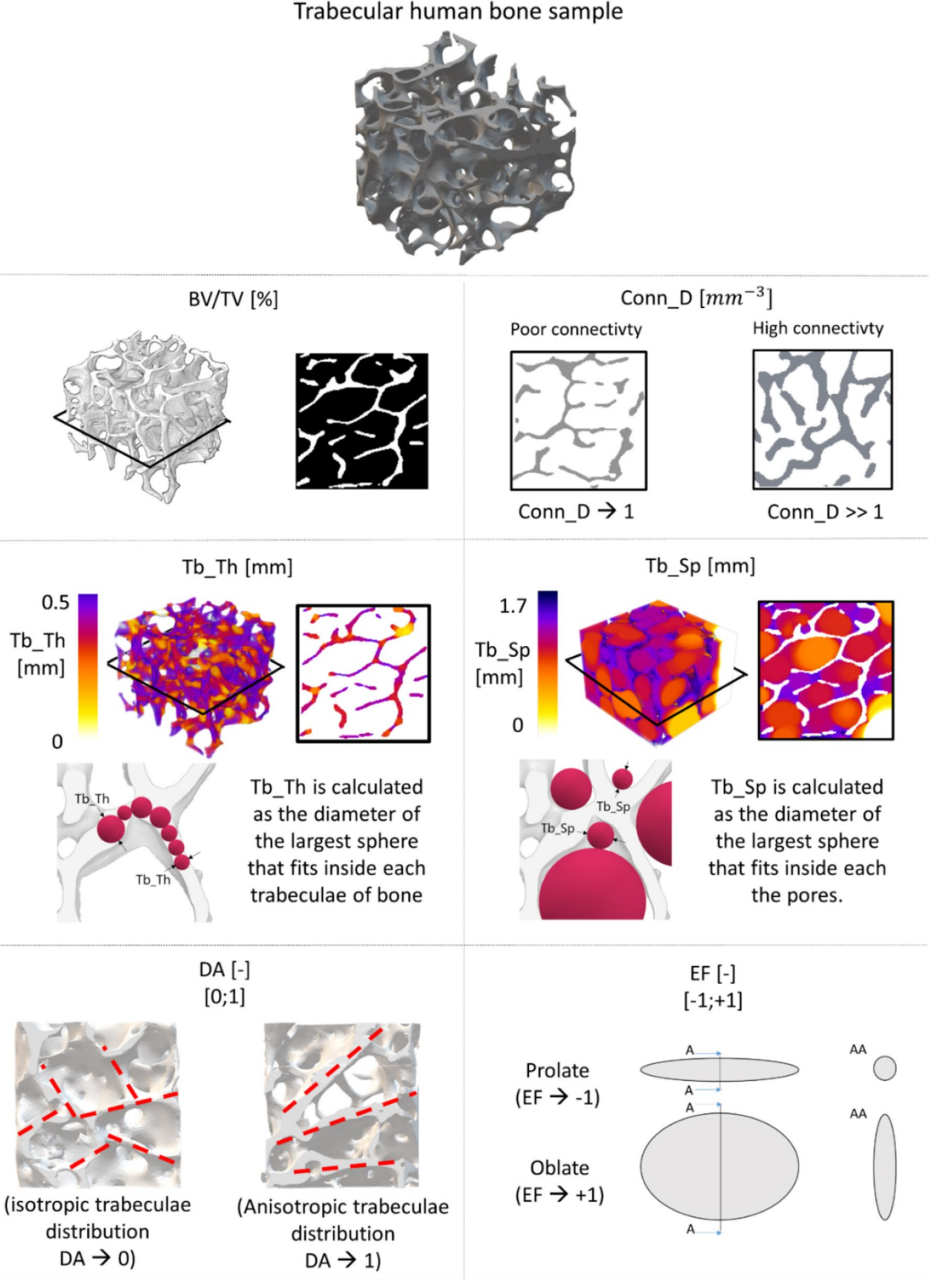
Morphometric parameters: bone density (BV/TV), connectivity density (Conn_D), trabecular thickness (Tb_Th), trabecular spacing (Tb_Sp), degree of anisotropy (DA) and ellipsoid factor (EF)

The measurement of morphometric parameters is carried out in BoneJ software, plugin of ImageJ [37] (Fig. 2). The evaluation of morphometric parameters includes both the measurement of the traditional ones [21], such as bone volume density (BV/TV) and average trabecular thickness (Tb_Th), trabecular spacing (Tb_Sp) and architectural bone parameters related to bone tissue organization such as connectivity density (Conn_D), degree of anisotropy (DA) and ellipsoidal factor (EF) [27].

Bone volume density is the average bone density inside the volume of interest, i.e. the cubic sample:

1 $$Bone\;volume\;density\;(BV/TV)=\frac{Bone\;volume(BV)}{Total\;volume(TV)}\times100\;\lbrack\%\rbrack$$

where *BV* is the volume of bone, while *TV* is the theoretical entire volume given by the sum of the volume of bone and the volume of void. Average BV/TV in osteoporotic patients is around 15–30% [11].

Tb_Th is calculated based on the model by Hildebran et al. [38, 39], and it is defined as the average dimension of trabeculae. Tb_Th is calculated as the diameter of the largest sphere that fits inside each trabeculae of bone. The algorithm requires binarization of the 3D image. Tb_Th ranges from 200 µm up to 400 µm in healthy patient: Nevertheless, Tb_Th values highly depend on anatomical location of the bone [40]. A colour map of trabecular thickness distribution in each sample is collected to be compared with the Tb_Th average value to evaluate its distribution inside the entire sample.

Tb_Sp is defined as the average dimension of pores, and it is calculated as the diameter of the largest sphere that fits inside binarized black background.

Conn_D describes the average number of trabeculae connected per unit volume, and it is based on the adaptation of Euler number (χ) [22].

2 $$Connectivity=1-(\upchi +\Delta\upchi )$$

3 $$Connectivity\;density\;(Conn\_D)=\frac{Connectivity}{Total\;Volume(TV)}\;\lbrack{mm}^{-3}\rbrack$$

where χ is the Euler characteristic and $\Delta\upchi$ is a correction term the get a more accurate estimation of the connectivity inside the whole network.

DA index describes the anisotropy orientation of the architectural arrangement of trabeculae. Anisotropy index ranges from 0 to 1: 0 stands for highly isotropic orientation of trabeculae, while 1 stands for highly anisotropic orientation. The quantitative description of structural anisotropy used the Mean Intercepts Length (MIL) method [41]. The output dots plot provides a visualization of the MIL points cloud which is described by a fitted ellipsoid, which axis elongation visually described the global anisotropy of the structure: The stretched is the ellipsoid; the higher is the degree of anisotropy in the trabeculae arrangement.

4 $$Anisotropy\;index\;(DA)=1-\frac{1/c^2}{1/a^2}$$

where *c* and *a* are the extreme radii of the ellipsoid fitted on the MIL points (a ≤ b ≤ c).

EF quantifies the plate-like or rod-like nature of each sample [27]. Firstly, ellipsoid fitting inside the trabeculae is measured to quantify the local nature of each trabecula (Eq. 5); thereafter, the global average value of EF results from the average of local EF measurements.

5 $$a \le b \le c, EF=\frac{a}{b}-\frac{b}{c}$$

where *a*, *b*, and *c* parameters are the three semi-axis length of each ellipsoid.

EF ranges from -1 up to + 1: The former indicates a plate-like bone (prolate shape), the latter stands for a rod-like bone (oblate shape). EF index is calculated considering an overall filling percentage equal to 90%. The algorithm implemented in BoneJ fills the BV with ellipsoids: highly prolate ellipsoids present one short axis and two short axes, while highly oblate ellipsoids show two long axes and one short axis [27]. Input constraints for the calculation of morphometric parameters result from an optimization analysis, and they are detailed in Appendix A.

### Mechanical testing: computational analysis

**Fig. 3:**
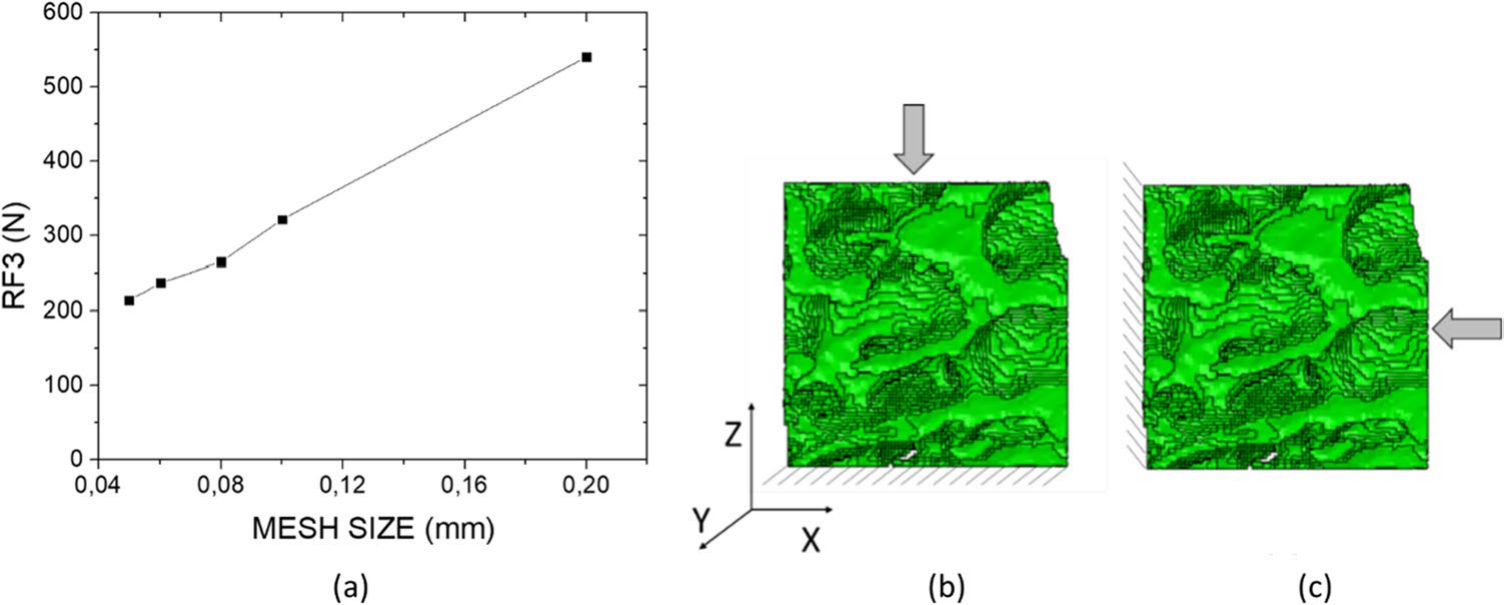
Mesh convergence analysis. Representative mesh convergence for OS_4: Similar results are obtained for all samples (**a**), boundary conditions to evaluate the apparent longitudinal stiffness (AL_STIFF) (**b**) and apparent transversal stiffness (AT_STIFF) (**c**)

FEA is carried out in Abaqus (v2017, SimuliaTM, Dassault, Germany). A shrink wrap mesh of linear hexahedral elements (C3D8) is adopted in Hypermesh software (Hypermesh v2019, Altair HyperWorks, United States). Brick elements are chosen since they are strongly suggested for models which involve bone tissue. The chosen element size to guarantee mesh convergence for the global model is set to 0.05 mm (Fig. 3a). Jacobian is equal to 0.8 [42]. Isotropic linear elastic material model is chosen with Young modulus and Poisson ratio equal to 1 GPa and 0.25, respectively [43]. Linear elastic homogeneous material behaviour is the most adopted in the literature, and it described the fragile behaviour of bone [4, 8]. The same Young modulus is used for all samples, regardless the physio-pathological state of bone: This choice, in spite of the clarifications below reported, may be considered as a limitation of the current work, and it may cause some errors in the real measurement of stress values. Despite it is well known that at such scale inhomogeneity of mechanical properties may occur [44], the current work does not focus on local inhomogeneities, and same Young modulus is considered for all samples; indeed, the current study aims to isolate and investigate the mesoscopic morphological, dimensional, and structural features of NOS and OS samples in order to evaluate their impact on the apparent mechanical response. Secondly, the binarization of the 3D set of image loses the grey values which may indicate regions with different mechanical properties, nulling the local inhomogeneity, which are not addressed in the current work. Two different sets of boundary conditions are applied: Firstly, the bottom side is fixed, and a displacement of 0.3 mm is applied longitudinally; secondly, one lateral side is fixed, and a transversal displacement along *x*-axis equal to 0.3 mm is applied. The choice of 0.3 mm is according to previous experimental tests [30]. Considering trabecular bone as a ‘cellular solid’ gives rise to the possibility to compute mechanical properties of the trabecular network in an efficient manner [34]. Apparent longitudinal stiffness and apparent transversal stiffness are calculated from the computational results (Fig. 3b–c). In particular, the former is calculated by considering the reaction force along *Z* and the longitudinal displacement along *z*-axis (i.e. AL_STIFF); the latter is calculated by considering the reaction force along *X* and the transversal displacement along *x*-axis (i.e. AP_STIFF).

### Statistical analysis

The data obtained from morphometric and computational analysis are analysed using JMP statistical software (JMP Statistical Discovery LLC, V17, US). Firstly, data normality is evaluated by means of Shapiro–Wilk test (*p*_value > 0.05 stands for normal data distribution); thereafter, proper statistical test is performed to compare each morphometric and computational variable for the two groups (Mann-Witney test and t-test for non-normal and normal data distribution, respectively). Graphical representation of mutual relationships among morphometric parameters and mechanical response in osteoporotic and non-osteoporotic samples is exhibited by means of interval plots (IC = 95%) for highlighting the effect of the factors on the variables, and confidential ellipses [45] (IC = 95%) for highlighting the correlation among the variables are adopted. Statistical significance is indicated with *p*_value < 0.05.

## Results

The study of mesoscale bone tissue level can have a direct impact into clinic with an advantageous discrimination of bone-related pathologies: indeed, DXA and cutting-edge investigation tools already implemented in clinics, CT imaging and MRI [13] are able to obtain images of the trabecular bone mesoscale morphological arrangement with high resolution compatible with trabeculae thickness [14, 16]. However, it is notable that higher resolution may require higher radiations for the patient: Therefore, future efforts in imaging field are needed to optimized the resolution without decreasing the safety of the CT procedure in terms of radiation safety. Imaging post-processing medical software, such as Mimics software (Materialise, Belgium), accept both CT and MRI medical images and provide the tools necessary for the segmentation and reconstruction of anatomical region of interest, such as bone sections, which, thereafter, can be then exported as stl. file format to be post-processed and evaluated by means of the methodology presented in the current work [13]. Depending on the morphological information to be acquired and reconstructed, proper CT and MRI resolution has to be chosen, and optimized reconstruction tools have to be employed [13]. Current results are therefore focused on mesoscale structure of human trabecular bone. Firstly, morphometric parameters are measured, and the statistical difference in morphometric parameters is investigated; later, the correlation among the morphometric parameters themselves is studied. Then, definition of mechanical response of each sample subjected to longitudinal and transversal displacement is also analysed. Finally, the mutual relationships among the morphometric parameters and the mechanical response are determined for the identification of parameters useful for the discrimination of bone samples based on their physio-pathological state.

### Evaluation of morphometric parameters of the bone samples

Morphometric parameters are measured for osteoporotic and non-osteoporotic samples (Table 2). All measurements are consequent to the definition of optimized input parameters (Appendix A).

**Table 2:** Morphometric parameters values for each sample. T_score values are provided by clinicians

Samples	Clinical condition	DA (-)	Conn_D ${(mm}^{3})$	BV/TV (%)	Tb_Th (mm)	Tb_Sp (mm)	Median EF (-)
OS_1	T_score < 2.5	0.66	3.1	16.9	0.184	0.897	-0.55
OS_2	T_score < 2.5	0.74	1.4	24.7	0.342	1.057	0
OS_3	T_score < 2.5	0.67	1.4	15.9	0.241	1.222	-0.15
OS_4	T_score < 2.5	0.7	2.3	28.3	0.375	1.147	-0.79
OS_5	T_score < 2.5	0.59	5.8	15.8	0.158	0.752	-0.52
OS_6	T_score < 2.5	0.71	4.1	20.4	0.224	0.772	-0.77
OS_7	T_score < 2.5	0.53	5.6	17.8	0.200	0.767	-0.66
NOS_1	T_score > 2.5	0.67	3.7	34.1	0.303	0.618	-0.55
NOS_2	T_score > 2.5	0.61	4.0	23.7	0.223	0.797	0
NOS_3	T_score > 2.5	0.35	2.0	35.3	0.374	1.005	-0.11
NOS_4	T_score > 2.5	0.59	5.2	21.3	0.217	0.747	-0.74
NOS_5	T_score > 2.5	0.44	6.6	23.3	0.213	0.739	-0.69
NOS_6	T_score > 2.5	0.55	7.7	34.9	0.229	0.488	-0.75
NOS_7	T_score > 2.5	0.51	4.7	28.2	0.219	0.678	-0.76

Once assessed the data normality (Shapiro Wilk test, *p* value > 0.05, null hypothesis of data normality accepted), t-test is performed for each morphometric parameter, but EF index. EF data present a non-normal distribution, for which a non-parametric test is employed (Mann–Whitney test).

In line from previous literature results [46], the BV/TV values reflect the pathological state of bone: Osteoporotic samples present a BV/TV lower than for non-osteoporotic samples with a high level of statistical significance (*p*_value < 0.05). In details, the average values of BV/TV decreases from 28.7% to 19.9%.

Tb_Sp values describe the results obtained from BV/TV: The more dense is the samples, the largest is the dimension of inner pores. Unilateral alternative hypothesis consists in observing the superiority of Tb_Sp values in OS samples in comparison with NOS. The analysis confirms that osteoporotic samples owns a Tb_Sp higher than for non-osteoporotic samples (*p*_value < 0.05). More specifically, the average values of Tb_Sp increase from 0.724 to 0.945 mm.

**Fig. 4:**
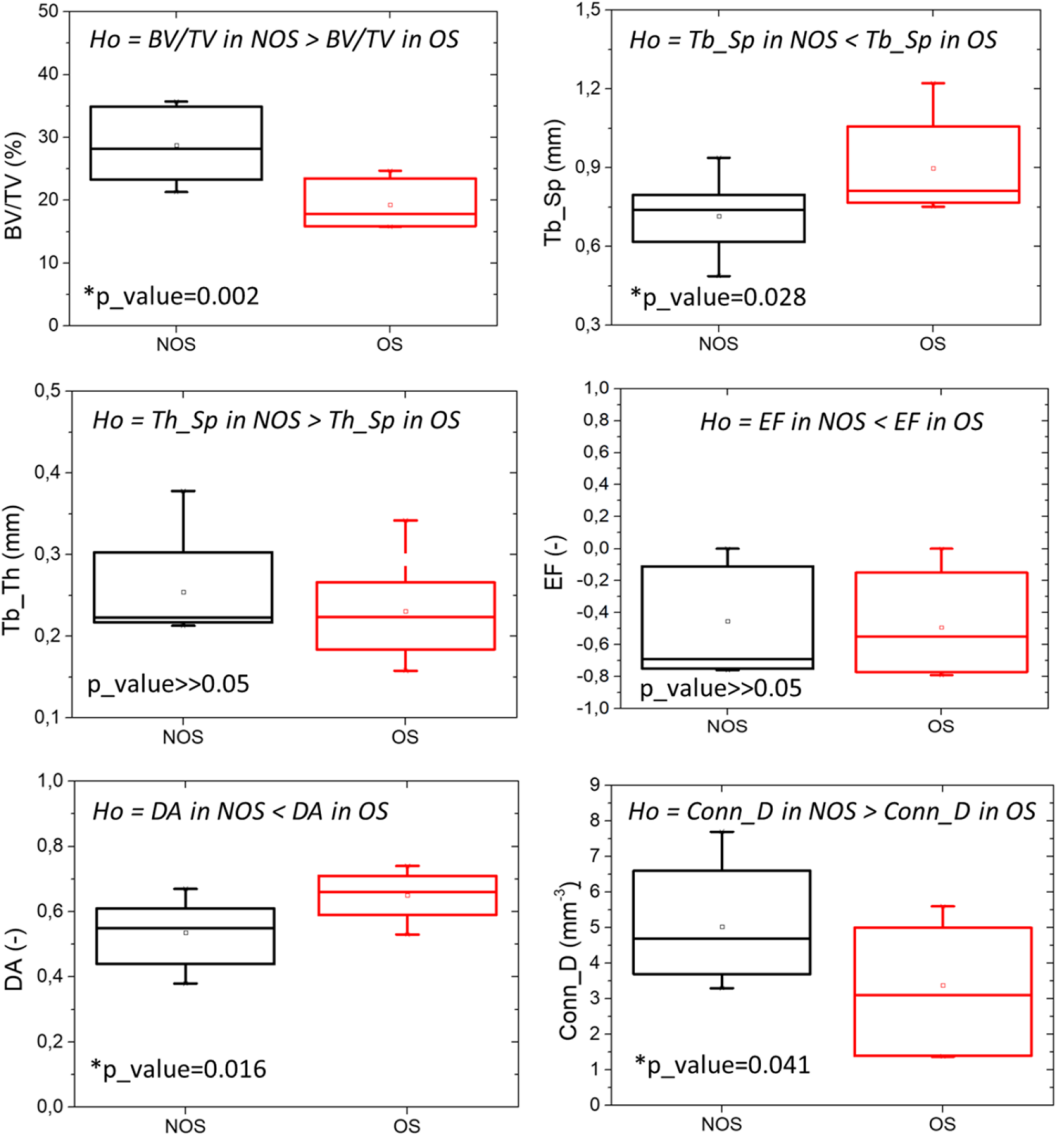
Statistical comparison of morphometric parameters between osteoporotic and non-osteoporotic samples. Specific null hypothesis is indicated for each *t*-test. Statistical significance is highlighted with asterisk (*p_value < 0.05)

**Fig. 5:**
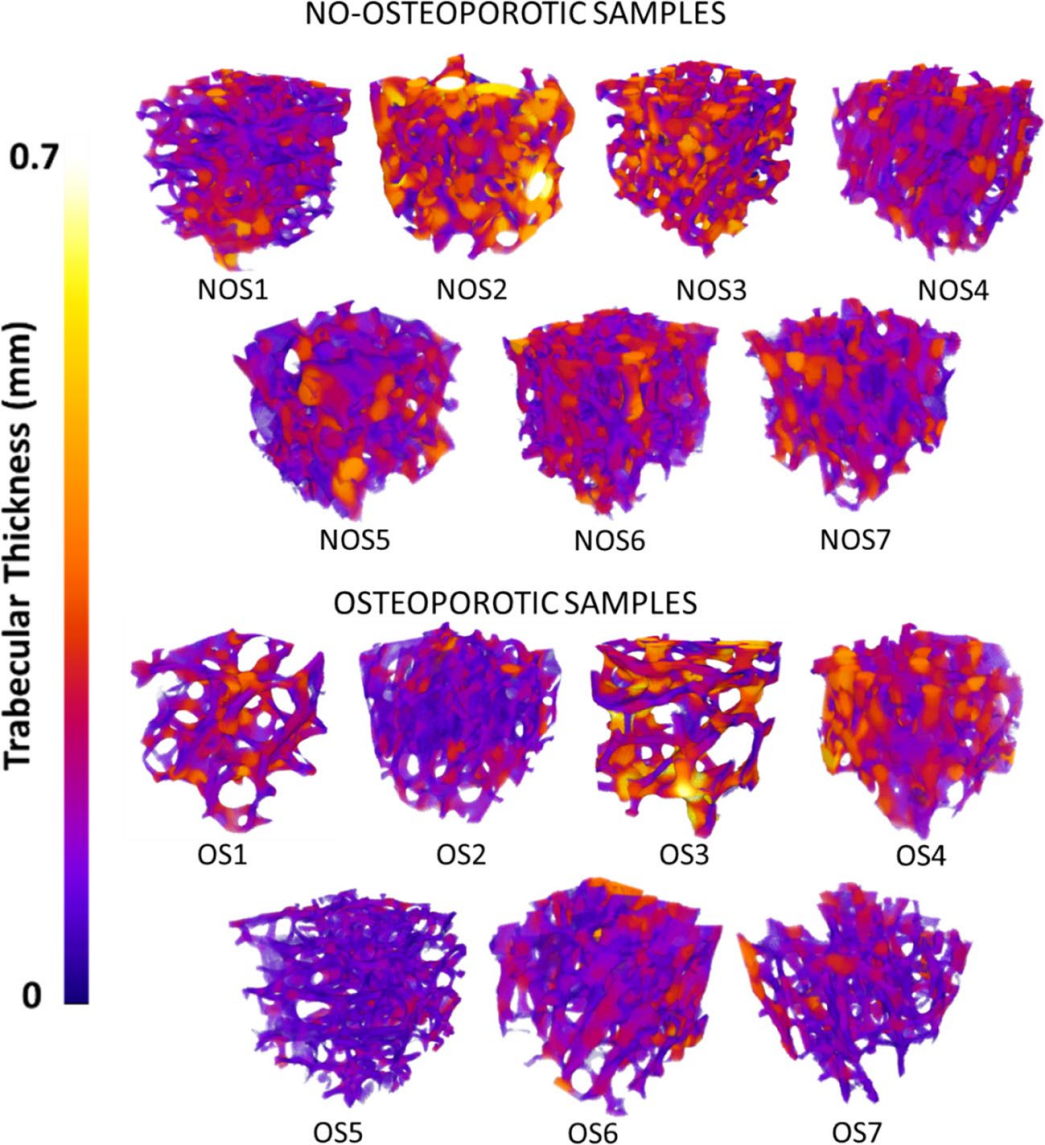
Colour map of trabecular thickness for each sample. Minimum trabecular thickness equal to 0 mm (dark purple colour) and maximum trabecular thickness equal to 0.7 mm (yellow-white colour)

Tb_Th average measurements present no statistical significant difference among osteoporotic and non-osteoporotic specimens (*p*_value > 0.1). Average value of Tb_Th are equal to 0.246 mm and 0.254 mm, for OS and NOS, respectively. The average values of Tb_Th are associated with the corresponding colour map of trabecular thickness with the aim to evaluate whether the average value of Tb_Th is the correct description of the trabecular thickness values inside the entire sample (Fig. 4). Due to the high variability in bone morphological arrangement, it may happen that the average values of Tb_Th is strongly affected by the present of only few large trabeculae: Therefore, the analysis of single trabeculae thickness could be useful, and a 3D colour map allows a correct interpretation of the measurement of Tb_Th (Fig. 5). It is worth noting that, on the contrary of expectation, OS_3 which should exhibit thinner trabeculae presents one of the thickest trabeculae among the analysed samples: However, this is a local result consequent to the high variability of samples, and it is associated with a large Tb_Sp. The interpretation of the only value of Tb_Th could have been misleading if not accompanied by a clear analysis of its distribution along the specimen and evaluation of different morphometric parameters: That is the reason why a parallel analysis of both values of Tb_Sp and Tb_Th should be carried out.

EF for osteoporotic and non-osteoporotic samples present no statistical significance (*p*_value > 0.05). Regarding the plate-like or rod-like nature of the samples, osteoporotic samples exhibit a more rod-like features (EF [µ ± σ] = -0.49 ± 0.3) contrary to non-osteoporotic samples in which a slightly decrease in EF value is registered (EF [µ ± σ] = -0.45 ± 0.35). Nevertheless, EF for osteoporotic and non-osteoporotic samples present no statistical significance (*p*_value > 0.05).

Concerning the 3D spatial orientation of trabeculae, DA values present statistical significant difference among NOS and OS samples (*p*_value < 0.05). DA index for osteoporotic samples indicate that trabeculae are oriented mainly along longitudinal direction, while non-osteoporotic samples own trabeculae oriented in a more isotropic way.

Concerning the architectural trabeculae arrangement, trabeculae are less interconnected in osteoporotic than in non-osteoporotic samples: Conn_D increases from 3.4 to 4.8 mm^−3^. The unilateral null hypothesis in the t-test consisting in assessing whether Conn_D in NOS group is significant greater than in OS group is confirmed by a *p*_value < 0.05.

### Evaluation of mutual relationships among morphometric parameters

**Fig. 6:**
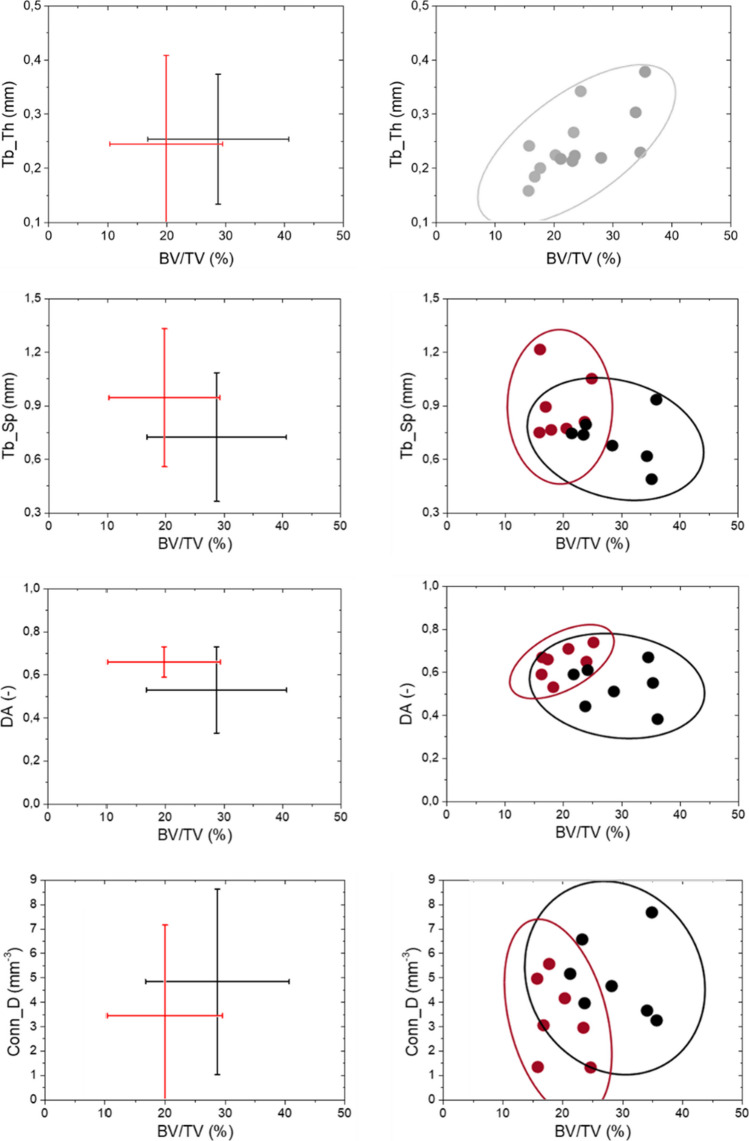
Interval plot and ellipsoid graphs for the representation of relationship among bone density and Tb_Th, DA and Conn_D. Interval plot reported µ ± 2σ. Confidential ellipses group data considering a confidential level equal to 95%

Results related to the relation among morphometric parameters are reported in Fig. 6. Firstly, the results are represented in interval plots with the aim to report the interval of each parameter respect to BV/TV, which is the most adopted morphometric parameters in clinic, also available from DXA investigation. Then, interval plots of mutual relationships among fabric bone tissue properties (DA, Conn_D and EF) are analysed.

Interval graphs allow the definition of variability for pair selected parameters (i.e. ± 2σ). The rectangular shape defined by the two lines delimits two possible areas in which osteoporotic and non-osteoporotic samples can be found with a percentage of 95% [47]. It is worth reminding that these preliminary analyses do not consider the interdependence between the chosen morphometric parameters; thus, the possible areas do not consider the correlation between the y_1_-y_2_ variables. Accordingly, additional investigations consisting in the definition of confidential ellipses for both osteoporotic and non-osteoporotic samples provide a graphical representation of the mutual relationships among pairs of morphometric parameters. The maximum axis of ellipses provides the correlation between the investigated morphometric parameters. In the event that one of the two graphed parameters is not statistically significant for the two groups (Fig. 4), all the samples are grouped in a unique sample in the evaluation of confidential ellipses. For instance, Tb_Th and EF are not statistically significant morphometric parameters, and when considered them, all fourteen bone samples have to be considered as a unique group for the analysis of confidential ellipses, and, consequently, only one ellipse is graphed.

Regardless the bone physio-pathological condition, Tb_Th is positive correlated with BV/TV (*R* = 0.65, IC = 95%, *p*_value < 0.05): denser samples exhibit higher Tb_Th values. Confidential ellipse points out the positive linear correlation between the morphometric parameters.

BV/TV is poor correlated with Tb_Sp (*R* ≈ 0 and *R* = -0.21 for OS and NOS samples, IC = 95%). From interval plots, it is clear that data’s variability is similar between OS and NOS samples. In spite of the presence of a quite relevant intersection area between the two ellipses, the available data provide two distinct areas of plane in which or osteoporotic or non-osteoporotic samples are individuated.

Concerning the relationship between DA and BV/TV, it is evident that the grater is the BV/TV, the lower is the DA in trabeculae bone arrangement, i.e. the arrangement is more isotropic. The intersection area of ​​the two ellipses coincides almost with the entire OS ellipse which samples are more similar each other than in NOS group: The available data indicates that OS samples are mainly grouped in the upper-left side of the graph, which means lower BV/TV and high DA, and two areas of plane can be distinguished for the two groups.

BV/TV presents positive linear correlation with Conn_D (*R* = 0.21 for OS and NOS, IC = 95%). The greater is BV/TV, the higher the Conn_D. From observation of confidential ellipses’ representation, a clear separation between NOS and OS samples is pointed out by. The two ellipses point out two areas which are clear distinct between NOS and OS samples, providing promising data for the discrimination of bone sample by considering its physio-pathological state: Indeed, OS samples are confined in the lower left area of the graph (i.e. low BV/TV and low Conn_D), while NOS samples are distributed in the upper right part of the graph (i.e. high BV/TV and high Conn_D).

**Fig. 7:**
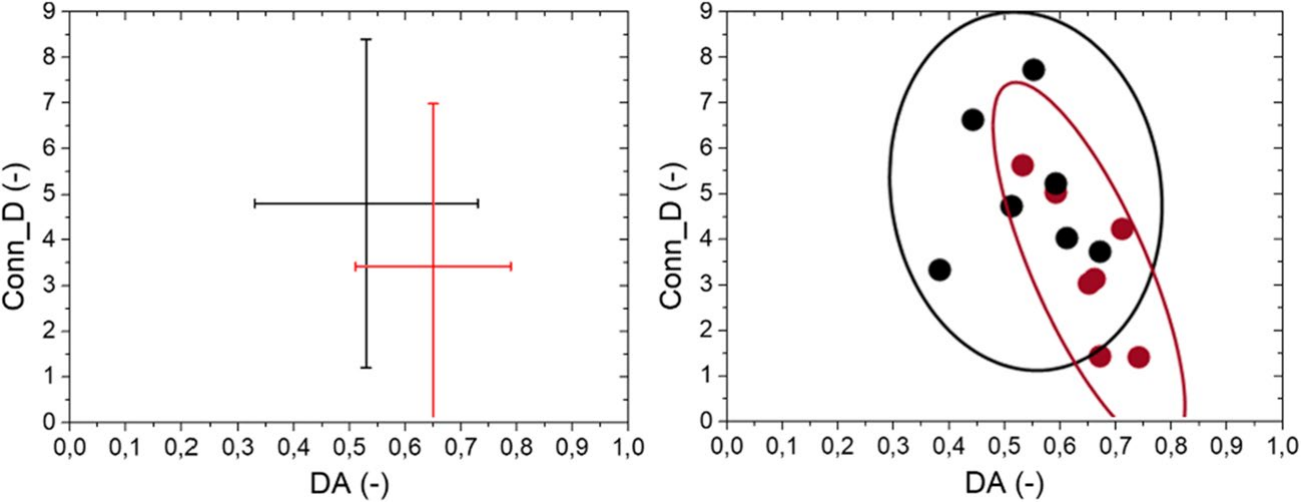
Mutual relationships between DA and Conn_D. Interval plot reported µ ± 2σ. Confidential ellipses group data considering a confidential level equal to 95%

Secondly, Fig. 7 reports the mutual associations between Conn_D and DA, which are the morphometric parameters related to the architectural arrangement of bone. From interval plots, the high variability of DA for NOS samples is evident. On the contrary, OS group exhibits lower variability in term of DA and higher in term of Conn_D. Considering the physio-pathological condition of bone, the correlation between Conn_D and DA is weak for NOS (R ≈ 0, IC = 95%), and it is strong for OS (R = -0.76, IC = 95%): The former result is consequent to the high variability inside the NOS group, the latter result demonstrates the negative relation between DA and trabecular connectivity.

**Fig. 8:**
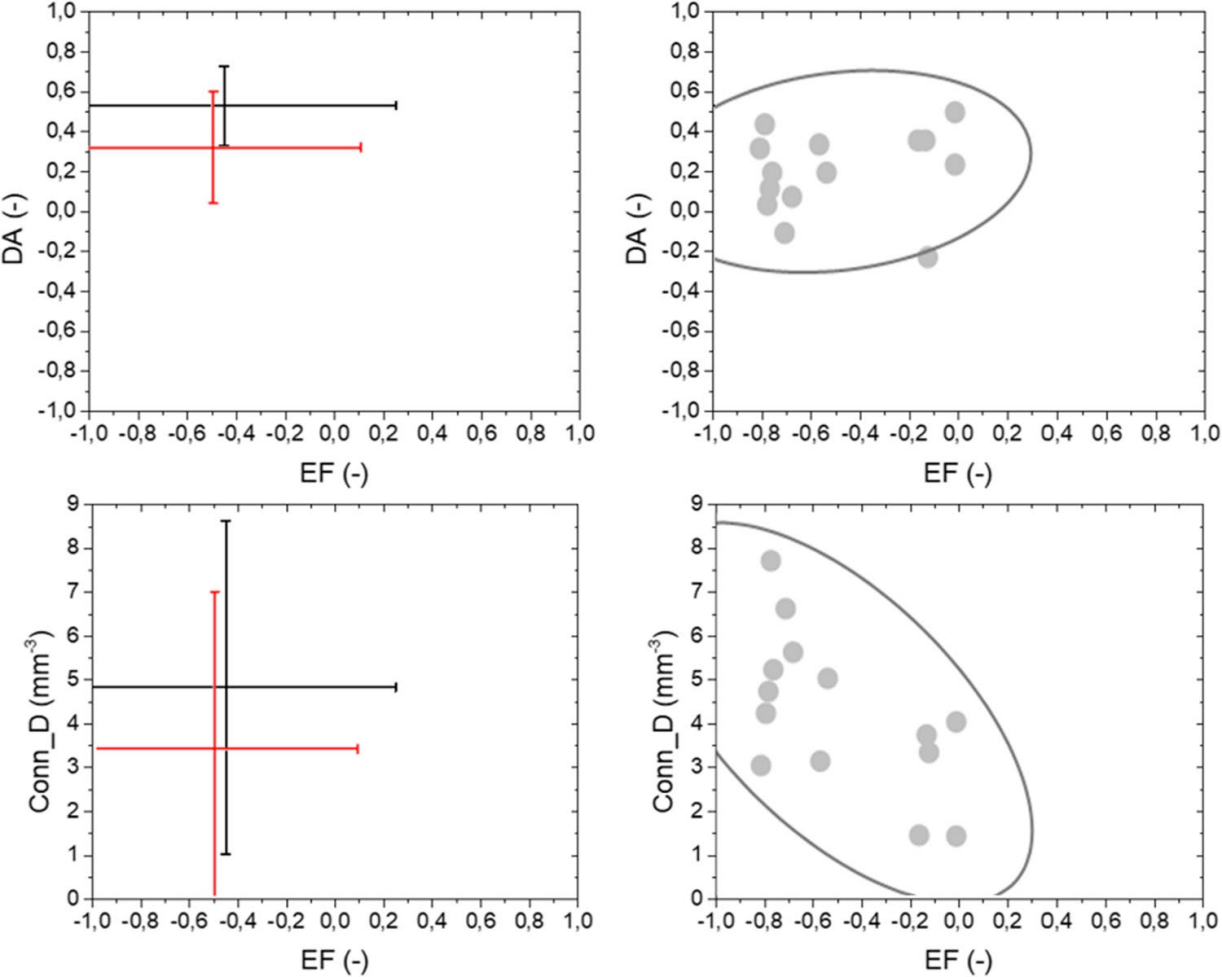
Mutual relationships between EF and fabric tissue parameters (DA and Conn_D). Interval plot reported µ ± 2σ. Confidential ellipses group data considering a confidential level equal to 95%

Finally, Fig. 8 summarizes mutual relationships among morphometric parameters related to the architectural arrangement of bone with EF. Regardless of the physio-pathological condition of bone, no relevant correlation is measured between EF and DA (*R* = 0.18, *IC* = 95%). As reported from the confidential ellipse, some data are close each other, while other data are outliers, e.g. #NOS2.

EF and Conn_D exhibit a negative correlation in their relationship, respectively (*R* = -0.62, *p*_value < 0.05).

### Evaluation of mechanical response of the bone samples

AL_STIFF and AT_STIFF are determined from FEA by considering the reaction force and the displacement along the longitudinal and transversal direction, respectively (Annex A-Figure A1). It is worth highlighting that bone stiffness strongly depend also on the percentage of components, such a collagen, hydroxyapatite; however, as detailed in Section 2.5, the current work adopts the same Young modulus for all samples, and it aims to focus on the special structural arrangement in order to evaluation its impact on the mechanical response [48].

Results are summarized in Table 3. Apparent stiffness measurements are consequent from the architectural arrangement of trabeculae which is different in each sample. Apparent stiffness values are normalized on length and apparent of the sample. Once assessed the data normality (Shapiro Wilk test, *p*_value > 0.05), *t*-test is performed for each morphometric parameter. Null initial hypotheses consist in the superiority of both NOS stiffness parameters.

**Table 3:** Apparent longitudinal stiffness (AL_STIFF) and apparent transversal stiffness (AT_STIFF) determined by means of computational analysis. Isotropic linear elastic material model is set (E, v). Average values of AL_STIFF and AT_STIFF based on the pathological state is reported as µ ± 2σ. (a) The high value of AT_STIFF is consequent to the high DA which characterizes the samples: The remained trabeculae are mainly oriented in the XY direction. (b) #NOS4 is among the oldest NOS patients, and low values of AL_STIFF and AT_STIFF, despite the T_score, can be hypothesised to be linked to the age of patient

Samples	Clinical condition	E (GPa)	v	AL_STIFF (N/mm^2^)	AT_STIFF (N/mm^2^)
OS_1	OS	1	0.25	104	140
OS_2	OS	1	0.25	104	251^(a)^
OS_3	OS	1	0.25	110	189
OS_4	OS	1	0.25	142	71
OS_5	OS	1	0.25	41	59
OS_6	OS	1	0.25	95	32
OS_7	OS	1	0.25	105	26
NOS_1	NOS	1	0.25	349	332
NOS_2	NOS	1	0.25	152	188
NOS_3	NOS	1	0.25	319	376
NOS_4^(b)^	NOS	1	0.25	81^(b)^	62^(b)^
NOS_5	NOS	1	0.25	134	93
NOS_6	NOS	1	0.25	281	190
NOS_7	NOS	1	0.25	226	142

**Fig. 9:**
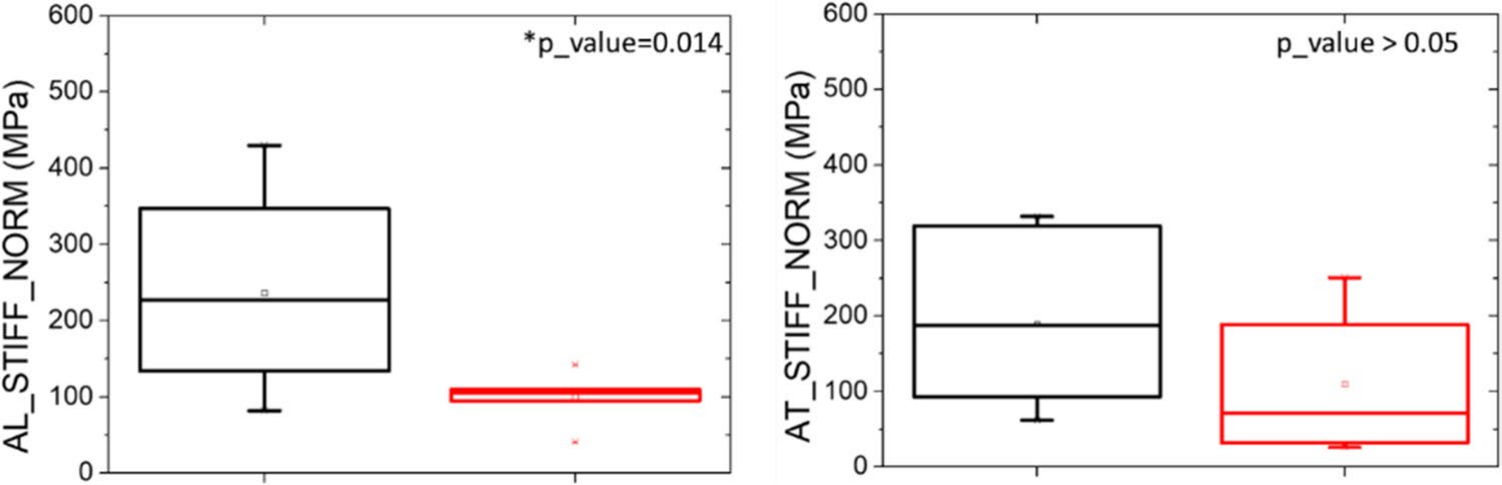
Mechanical response in term of apparent longitudinal (AL_STIFF) and transversal stiffness (AT_STIFF). Values were normalized on sample’s size (_NORM). Statistical significance is highlighted with asterisk (*p_value < 0.05)

The greater is the stiffness along specific direction, the greater is the strength of the architecture along that axis. Osteoporotic samples own AL_STIFF and AT_STIFF lower than for non-osteoporotic samples, confirming the null hypothesis (Fig. 9). The average value of AL_STIFF increases from 100 up to 236 N/mm^2^, for osteoporotic and non-osteoporotic samples, respectively. The average value of AT_STIFF increases from 110 to 190 N/mm^2^, for osteoporotic and non-osteoporotic samples, respectively. AL_STIFF index is statistically significant for the two groups (*p*_value < 0.05), while at first sight, AT_STIFF parameter looks not statistical significant for the two groups (*p*_value > 0.05). Nevertheless, special attention should be focused on NOS4. The sample was harvested from the oldest NOS patients, and low values of AL_STIFF and AT_STIFF are registered: According to T_score, osteoporosis does not occur, but the age of patient and possible additional pathologies strongly affects the mechanical evaluation. NOS4 should be considered an outlier in its own group, making the new average values equal to 243 N/mm^2^ and 210 N/mm^2^ for AL_STIFF and AT_STIFF, respectively. In case of its removal from the statistical analysis, AT_STIFF of OSs is significant lower than NOS group (*p*_value < 0.05).

Secondly, the high AT_STIFF of OS2 can be explained by the fact that trabeculae are oriented in an anisotropic way and are mainly oriented in the horizontal direction (DA = 0.74): Therefore, the stiffness is much greater in the transversal direction.

### Relation between morphometric parameters and mechanical response

**Fig. 10:**
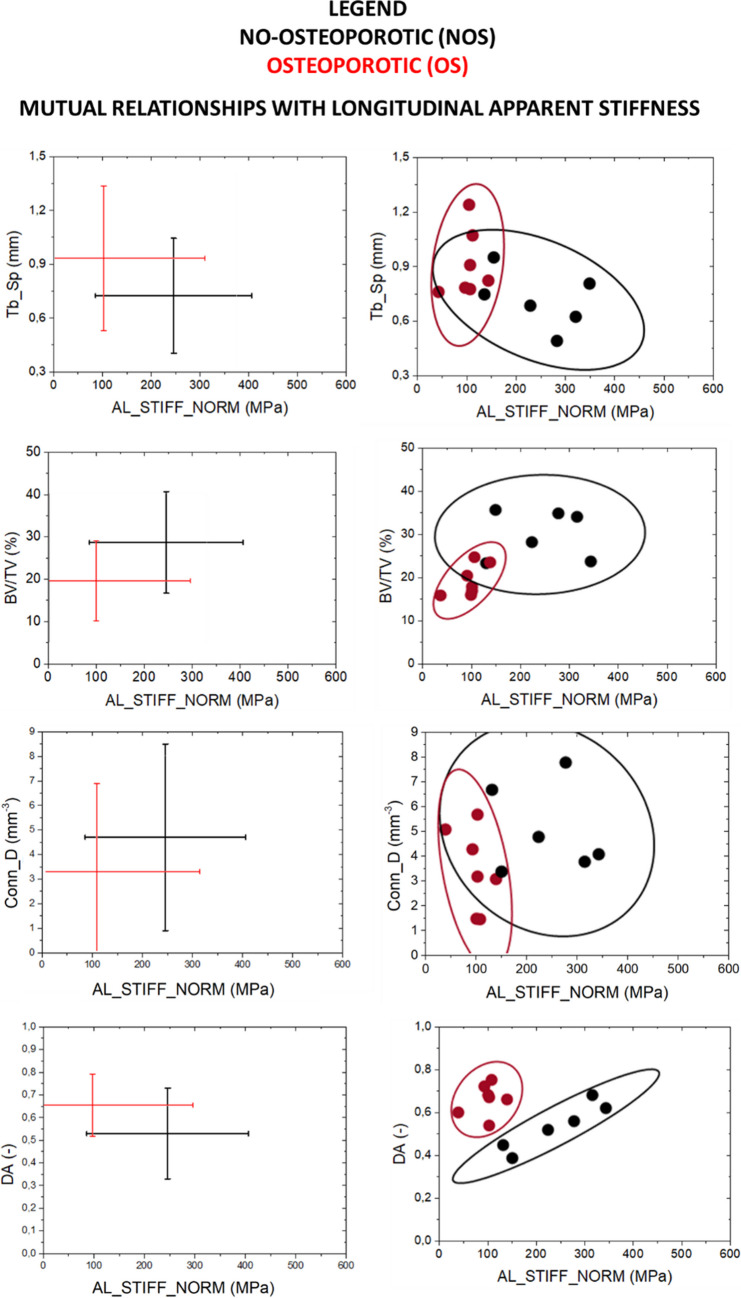

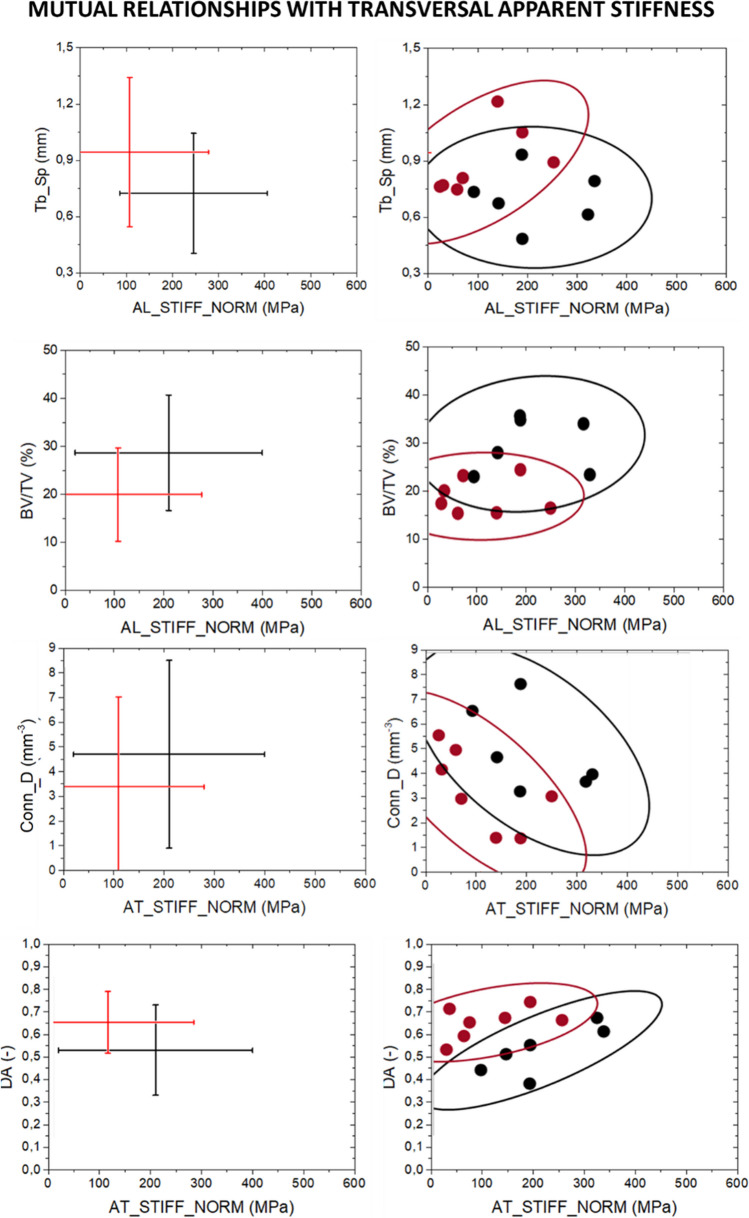
*XY*-interval plots for the statistical significant morphometric parameters related to mechanical response (AL_STIFF and AT_STIFF). Interval plot reported µ ± 2σ. Confidential ellipses group data considering a confidential level equal to 95%

From the abovementioned results, it is evident that mechanical response must be discussed in correlation with bone morphometric parameters. Mutual relationships between each morphometric parameters and global mechanical response at mesoscale are summarized in interval plots (Annex A-Figure A2). For OS and NOS groups, the variability in AT_STIFF is more than 50% higher than in AL_STIFF: Interval plots point out areas where NOS and OS samples can be observed (µ ± 2σ). However, as anticipated in Section 3.2, no dependence between the variables is considered in the interval plots: for a graphical representation of the mutual relationships among morphometric parameters, confidential ellipses for statistical significant morphometric parameters (Fig. 10). Relationships between AL_STIFF and AT-STIFF with the most statistically significant morphometric parameters, i.e. DA, BV/TV, Conn_D and Tb_Sp, are reported in Fig. 10. Sample NOS4 is evaluated as above reported, and it is removed in the mutual relationships analysis as it is an outlier, and its presence would mislead the evaluations.

Firstly, similar relationships between NOS and OS group respect both AL_STIFF and AT_STIFF are evident.

For Tb_Sp, when AT_STIFF and AL_STIFF increases, the spacing among trabeculae decreases. The correlation between Tb_Sp and AL_STIFF shows that the lower is the former parameter, the higher is the latter one: The OS samples are confined in the left part of the graph (low AL_STIFF values), and NOS samples cover the right part of the graph without highlighting any strong correlation. Similar results for the mutual relationships with Tb_Sp and AT_STIFF. In both cases, the intersection areas of the two ellipses are quite large which makes necessary the analysis of mutual relationships with additional morphometric parameters.

BV/TV, as already reported in the literature [46], turns out to be crucial morphometric parameters for the discrimination of physio-pathological state of bone: The greater is BV/TV, the greater are both AL_STIFF and AT_STIFF. A positive correlation between average values of BV/TV and apparent stiffness measurements is pointed out in the interval plots. From confidential ellipse, it is evident the possibility to define two areas pf plane in which only NOS or OS samples are separated and included.

DA and Conn_D result to be the best morphometric parameters able to define different regions of plane in which or NOS or OS are present. The greater is the longitudinal and transversal stiffness, the higher is the connectivity of trabeculae which are characterized by an isotropic arrangement.

## Discussion

The existence of mutual relationships between trabecular structures and mechanical response have been hypothesised in the literature [5, 11, 49]: However, current researches are still ongoing to provide useful indicators for a clear discrimination of bone state according to its physio-pathological state. The current work aims at shedding light on the correlations between the mesoscale features, i.e. morphometric parameter and the mechanical response consequent to the architectural arrangement of trabeculae which is strongly affected by physio pathological state of bone. In contrast to the microscale and nanoscale levels of analysis, where current findings face challenges in direct clinical application, mesoscale studies offer a resolution that aligns with existing clinical resolutions. Consequently, research conducted at the mesoscale holds greater potential for straightforward application in clinical settings. The identification of correlations between mesoscale bone features and mechanical response will have a direct impact into clinic providing additional information for the discrimination of physio pathological state of bone.

Currently, DXA scans, already implemented in clinics, provide information on bone density and WHO provides well-established indexes for the definition of osteoporosis (i.e. T-score, z-score) [9, 10]. The reason of considering more morphometric parameters instead of the only common ones, i.e. BV/TV and Tb_Th, is that the complex bone structure and their high variability cannot be firmly described by means of only few parameters [11, 12]. Indeed, the only bone density values may mislead the correct discrimination of bone physio-pathological state, especially at the beginning of the onset of the disease: Parameters related to fabric properties and bone morphological arrangement are crucial for the overall comprehension of bone structure at mesoscale and for the early diagnosis of bone-related diseases.

From the results, osteoporotic samples differ above all in bone density, trabecular spacing, trabeculae interconnectivity and degree of anisotropy. As expected, osteoporosis is a bone disorder that induces a decreasing in bone density, and our results confirmed that [34]. According to the proposed analysis, trabecular spacing, despite not analysed in clinic, resulted to be a useful parameter for the discrimination between osteoporotic and non-osteoporotic samples. Regarding the fabric tissue properties, the connectivity among trabeculae decreases a lot in osteoporotic samples than non-osteoporotic ones, with a difference in the average values of Conn_D equal to 30%. Similarly, DA results to be a significant parameter for the discrimination of the physio-pathological state of bone: Osteoporotic trabeculae are oriented predominantly along one preferential direction leading to a higher anisotropy index than for non-osteoporotic samples. The number of connections among trabeculae and the way of their mutual positioning in the space resulted one of the most relevant hints for osteoporosis. Tb_Th and EF morphometric parameters provide not statistically significant difference between the two groups.

Osteoporosis strongly affects the mechanical strength of bone [46]. The adoption of Gibson-Ashby model [34] permits to study each bone volumes as a “black box” leading to a more efficient determination of apparent mechanical responses affected by the trabeculae 3D arrangement. Both apparent transversal and longitudinal stiffness decrease in osteoporotic sample: The lower mechanical response is accompanied by a lower Conn_D, higher Tb_Sp and DA and lower BV/TV (Fig. 10). The use of FEA helps in the definition of additional parameters (AL_STIFF and AT-STIFF) which depends on bone global mechanical response: This mechanical response is related to the way in which trabeculae are arranged in the 3D space. All these preliminary analyses point out that mutual relationships among morphometric parameters and mechanical response may vary according to the physio pathological conditions of bone.

Considering the aforementioned examinations, the preliminary results of the current research state that osteoporosis leads to a decreasing in bone density, increasing in trabecular spacing and degree of anisotropy and decreasing in the number of trabeculae interconnection coupled with a decreasing in mechanical response.

To date, beside BV/TV index, no specific values of the morphometric parameters are well-established considered as cut-off indices useful for the discrimination of bone samples considering its physio-pathological state. The analysis of confidential ellipses (IC = 95%) provides results useful for the definition of regions of plane in which or osteoporotic samples nor non-osteoporotic samples are included. Furthermore, from these graphs, an intersection area between the two ellipses is reported, indicating a region in which the diagnosis of pathology is difficult. Therefore, in such case, the possibility of examining additional graphs which include different morphometric parameters and apparent mechanical features can provide additional information for the improvement in bone-sample state description. Finally, the age should be considered as an additional parameter for a better comprehension of the morphometric features of bone, both osteoporotic and non-osteoporotic.

The analysis of mutual associations between bone parameters and mechanical response provides more information for the discrimination between NOS and OS samples, otherwise not always available from the only analysis of individual more studied parameters, i.e. BV/TV.

The major drawback of the current research regards the small sample size which may affect the statistical analysis leading to an increase in data variability, already highly affected by the extreme intrinsic variability of bone architectural arrangement. Though aforementioned limit, the current work aims to propose a preliminary approach useful to compare bone morphometric features for the improvement in the early diagnosis of osteoporosis and for the evaluation of mutual relationships among more bone parameters which are often underestimated. Certainly, the robustness of confidential ellipses will be strengthening with a greater sample size. Secondly, higher data variability is observed for NOS samples in comparison with OS group ones. This finding is consequent to the fact that the available samples are clinically described only by the presence of osteoporosis and non-osteoporosis: No additional medical information about any further bone-related pathologies are currently available. Consequently, in NOS group, there are bone specimens which may have different bone-related pathologies, but surely no osteoporosis disease. On the contrary, the OS group assembles only bone sample with diagnosed osteoporosis, with a consequent low variability in terms of differences among its samples. Finally, a possible drawback related to the current work concerns the need for high-resolution CT images necessary to appreciate and employ the obtained results into clinical field: Indeed, this fact could lead to clinical practice not being able to immediately use the current results. Therefore, further efforts may be needed to disseminate the clinical use of high-resolution CT equipment without decreasing radiation safety. In conclusion, to address the aforementioned drawbacks, multidisciplinary research collaborations are currently ongoing to obtain further additional human bone samples from femoral head to be investigated through the approach proposed in the present work.

## Conclusions

The current research aims at making clear the mutual relationships between bone morphometric parameters and mechanical response at mesoscale. The main outcomes of this research are summarized as follows:

- The use of a single morphometric parameter at mesoscale can often be misleading and does not allow for a correct definition of the physio-pathological condition of the sample.
- The concept of “cellular solids” allows the definition of quantitative information on global mechanical response, such as apparent transversal and longitudinal stiffness which strongly depend on architectural 3D arrangement of bone at mesoscale.
- BV/TV, Conn_, Tb_Sp and DA indices exhibit statistical significant difference between the two groups. From the mutual relationship analysis, they are considered as the most promising morphometric parameters for the discrimination between osteoporotic and non-osteoporotic state.
- BV/TV and Tb_Th are positive correlated: Their linear correlation presents a trend which can be exploited for hypothesizing a physio-pathological classification of additional bone samples.
- AL_STIFF and AT_STIFF are strongly related to bone density and connectivity density.
- Conn_D and DA result the best predictors of mechanical response of bone.
- Mutual relationships among morphometric parameters and mechanical response permit the definition of areas of plane which provide graphical information relevant for the discrimination of physio-pathological bone condition.

The main drawback of the current study regards the small dimension of samples size which inevitably affect the statistical analysis. Moreover, the high dispersion of data was even worsened by the intrinsic variability in bone trabecular arrangement. Further researches are ongoing to increase the samples size with the aim to increase the robustness of our data for a better discrimination of bone samples based on their physio pathological conditions.

## Supplementary Information

Below is the link to the electronic supplementary material.

Supplementary file1 (DOCX 691 KB)
