# Supplementary material for: Effect of trabecular architectures on the mechanical response in osteoporotic and healthy human bone
Source: Med Biol Eng Comput. 2024 Jun 1;62(11):3263–81. doi: 10.1007/s11517-024-03134-8 (PMC11485120; doi:10.1007/s11517-024-03134-8)
Supplement: Supplementary file 1 — Supplementary file1 (DOCX 691 KB) [file 11517_2024_3134_MOESM1_ESM.docx]

# Appendix A

The calculation of each morphometric parameters was preceded by an optimization of input parameters: following data summarize the adopted optimized parameters.

| Ellipsoidal factor (EF) | |
| --- | --- |
| Vectors | [50-100] |
| Sampling increment | 0.03 |
| Skeleton points per ellipsoid | [1-10] |
| Contact sensitivity | 50 |
| Maximum iterations | 50 |
| Maximum drift | 2 |
| Repetitions | 10 |
| Average over largest n ellipsoids | 1 |
| Seed points based on distance ridge | Flagged |
| Threshold for distance ridge | 0.05 |

**Table A1:** input parameters for ellipsoid factor measurement. The greater the number of vectors, the greater is the filling percentage. Proper value of vectors for each parameters was chosen to confirm a filling percentage greater than 90%.

| Degree of anisotropy | |
| --- | --- |
| Directions | 10000 |
| Lines per direction | 10000 |
| Sampling increment | 1.73 |

**Table A2:** input parameters for degree of anisotropy measurement


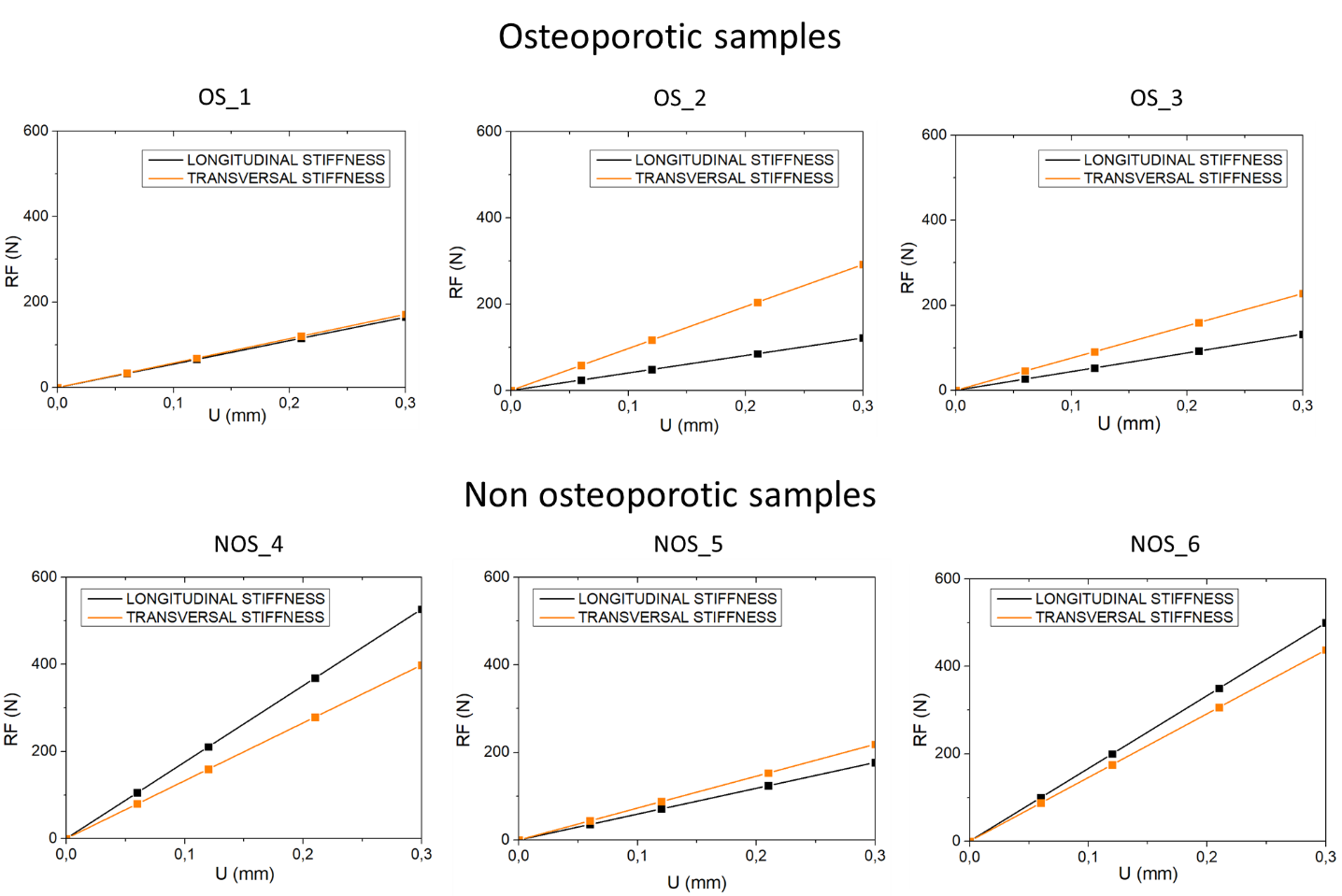


**Figure A1:** graphical representation of apparent stiffness defined for 3 NOS and 3 OS sample by means of FEA. Reaction force along z (RF3) over transversal displacement along z (U3) lead to the definition of AL_STIFF. Reaction force along x (RF1) over transversal displacement along x (U1) lead to the definition of AT_STIFF. All the values of apparent stiffness are normalized on sample areas and size. Similar analyis is performed for all samples.

**
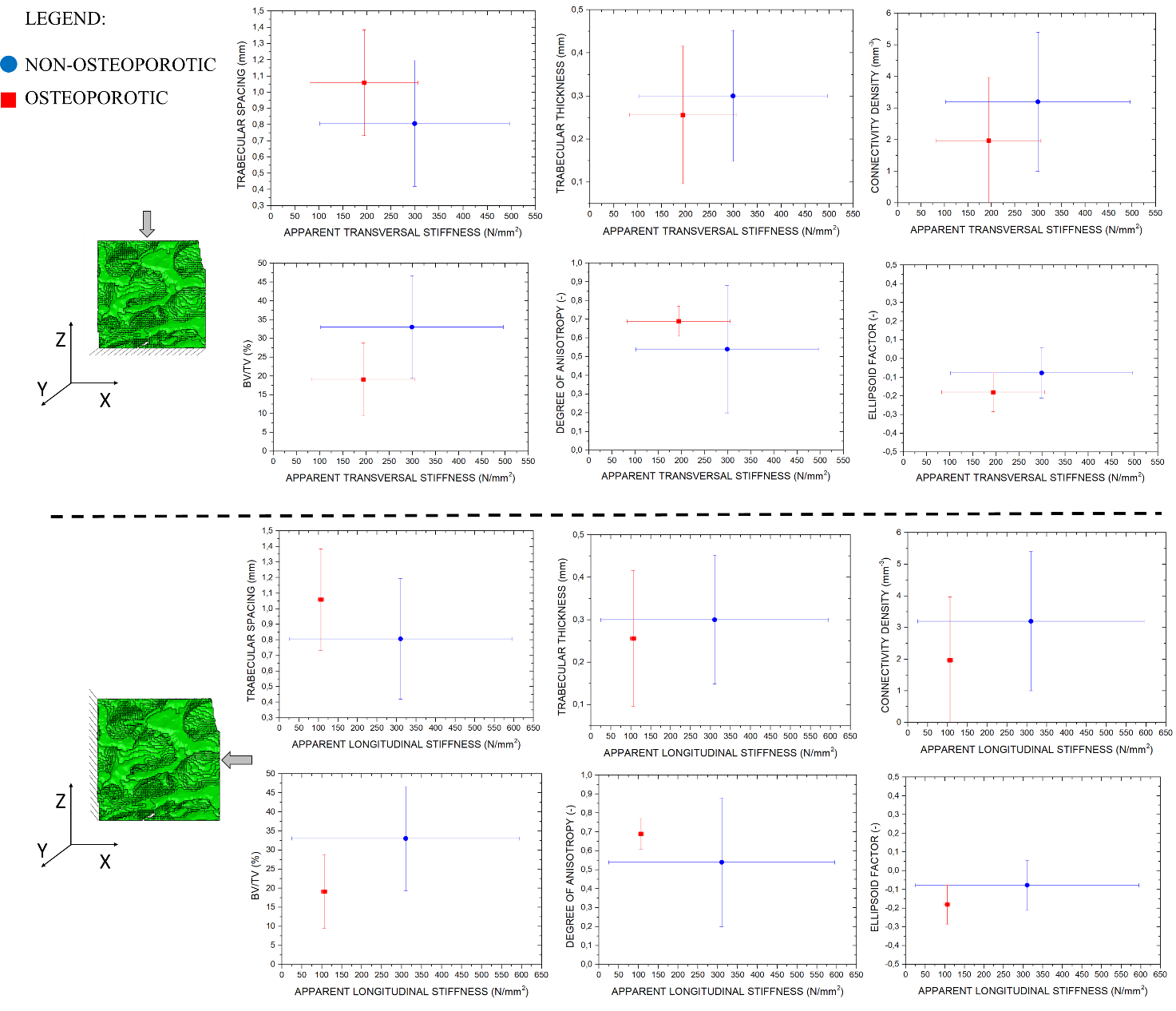
**

**Figure A2**: interval plots related to the relation among each morphometric parameter and global mechanical responses (apparent longitudinal stiffness and apparent transversal stiffness). Blue colour stands for non-osteoporotic samples and red-colour stands for osteoporotic measurements. In xy-interval plot µ ± σ are reported. Each point defines the average value of morphometric parameter of interest (i.e. µ of BV/TV and AL_STIFF); the error bars are equal to plus/minus standard deviation (i.e. ± σ).
